# Overexpression of CENPF correlates with poor prognosis and tumor bone metastasis in breast cancer

**DOI:** 10.1186/s12935-019-0986-8

**Published:** 2019-10-11

**Authors:** Jingbo Sun, Jingzhan Huang, Jin Lan, Kun Zhou, Yuan Gao, Zhigao Song, Yunyao Deng, Lixin Liu, Ying Dong, Xiaolong Liu

**Affiliations:** 1 Department of General Surgery, The Third Affiliated Hospital of Southern Medical University, 183 West Zhongshan Avenue, Guangzhou, 510630 Guangdong China; 2 Department of Metabolic Surgery, General Hospital of Guangzhou Military Command, Southern Medical University, Guangzhou, 510515 China; 3 Nursing Department, The Third Affiliated Hospital of Southern Medical University, 183 West Zhongshan Avenue, Guangzhou, 510630 Guangdong China

**Keywords:** CENPF, Breast cancer, Bone metastasis, mTORC1

## Abstract

**Background:**

Centromere Protein F (CENPF) associates with the centromere–kinetochore complex and influences cell proliferation and metastasis in several cancers. The role of CENPF in breast cancer (BC) bone metastasis remains unclear.

**Methods:**

Using the ONCOMINE database, we compared the expression of CENPF in breast cancer and normal tissues. Findings were confirmed in 60 BC patients through immunohistochemical (IHC) staining. Microarray data from GEO and Kaplan–Meier plots were used analyze the overall survival (OS) and relapse free survival (RFS). Using the GEO databases, we compared the expression of CENPF in primary lesions, lung metastasis lesions and bone metastasis lesions, and validated our findings in BALB/C mouse 4T1 BC models. Based on gene set enrichment analysis (GSEA) and western blot, we predicted the mechanisms by which CENPF regulates BC bone metastasis.

**Results:**

The ONCOMINE database and immunohistochemical (IHC) showed higher CENPF expression in BC tissue compared to normal tissue. Kaplan–Meier plots also revealed that high CENPF mRNA expression correlated to poor survival and shorter progression-free survival (RFS). From BALB/C mice 4T1 BC models and the GEO database, CENPF was overexpressed in primary lesions, other target organs, and in bone metastasis. Based on gene set enrichment analysis (GSEA) and western blot, we predicted that CENPF regulates the secretion of parathyroid hormone-related peptide (PTHrP) through its ability to activate PI3K–AKT–mTORC1.

**Conclusion:**

CENPF promotes BC bone metastasis by activating PI3K–AKT–mTORC1 signaling and represents a novel therapeutic target for BC treatment.

## Background

Breast cancer (BC) remains a leading cause of cancer related death in women across the globe [1]. In total, 60% to 75% of metastasis in BC leads to bone metastasis (BM) [2]. Bone metastasis impair the quality of life due to hypercalcemia, bone pain, fractures, nerve compression, a reduction in mobility, and reduced social function [3, 4]. When bone metastasis occurs, the disease enters an incurable stage, with a median survival time of only 2 years, and 5-year survival rates of 20% [5–8]. Controlling bone metastasis in breast cancer remains a problem in clinical practice.

Bone metastasis is a complex, multistage process that includes local invasion, intravasation, survival in the circulation, extravasation, and colonization [9, 10]. An array of pathogenic molecules mediate BC bone metastasis including parathyroid hormone-related protein (PTHrP), interleukin 8 (IL-8), and vascular cell adhesion molecule 1 (VCAM-1) [11–13]. Despite progress in the molecular basis of bone metastasis in BC, knowledge of the mechanisms underlying this process are required to identify targets for the prevention and treatment of BC.

The Centromere Protein F (CENPF) is a cell cycle-associated nuclear antigen that is expressed to low levels in G0/G1-cells and accumulates in the nuclear matrix during the S-phase, with maximal expression in G2/M-cells. CENPF was identified as a marker of cell proliferation in several human malignancies, including BC [14] and is overexpressed in hepatocellular carcinoma (HCC) [15] and other tumors [16]. Additionally, elevated CENPF expression contributes to unregulated cell proliferation in HCC [17]. It was recently shown that CENPF and FOXM1 are synergistic master regulators of prostate cancer malignancy and are prognostic indicators of poor survival and metastasis [18]. Furthermore, COUP transcription factor 2 (COUP-TFII) promotes metastasis in prostatic cancer (PC) through CENPF signaling [19].

In this study, we demonstrate that CENPF is a valuable prognostic predictor of BC. Bioinformatics and computational analysis demonstrated that CENPF regulates BC metastasis to bone through PI3K–AKT–mTORC1 signaling. And we have comfirmed this signaling by western blot. PI3K–AKT–mTORC1 signaling activation results in the increased secretion of PTHrP, and modification of the host osseous environment to promote osteoclast formation and bone colonization. Taken together, these findings provide novel insight into the mechanisms of bone metastasis in BC.

## Materials and methods

### ONCOMINE analysis

mRNA levels of CENPF in BC were determined through analysis of the ONCOMINE database (http://www.oncomine.org), a publicly accessible online cancer microarray database that facilitates the discovery of genome-wide expression analyses. A Students *t* test was used for the comparison of cancer specimens and normal control datasets. Fold changes were defined as 2 and a *p*-value ≤ 0.01 was deemed significant.

### Microarray analysis

CENPF gene expression data and the corresponding related clinical parameters were downloaded from the publicly available GEO website (http://www.ncbi.nlm.nih.gov/geo/) including GSE2034 and GSE5034. GSE2034 datasets contained 286 samples, including 180 lymph-node negative relapse free BC patients and 106 lymph-node patients that developed distant metastasis. GDS5306 dataset contained 19 HER2 + human BC brain metastasis patients and 19 HER2 + primary breast tumors.

### Prognostic survival analysis

Clinical prognostic analysis including the overall survival (OS) and relapse free survival (RFS) of CENPF were performed using the Kaplan–Meier method (http://kmplot.com/analysis/). Kaplan–Meier survival curves, log-rank *P*-values and HR with 95% confidence intervals were calculated and plotted in R using Bioconductor packages. Datasets with clinical prognostic information including GSE2034 and GSE39582 were used for prognostic survival analysis. mRNAs in all the datasets were divided into high expression (High) and low expression (Low) groups according to the mean values of CENPF expression.

### Gene set enrichment analysis (GSEA)

Gene set enrichment analysis (GSEA) was used to interpret the gene expression data by determining statistically significant differences in pre-defined gene-sets between biological states. In addition, GSEA can be used to identify the pathways that correlate to gene expression. To probe the biological mechanisms using GSEA software v2.1.0 (Broad Institute, MIT, Cambridge, MA, USA), a 32,619 (gene) × 39 (samples) expression matrix was employed. The predefined gene set ‘c2.all.v4.0.symbols.gmt’ is one of 7 major collections from the Molecular Signatures Database (MSigDB). A normalized enrichment score (NES) was calculated as the primary GSEA statistic. Gene sets were considered significantly enriched at predefined p-values and FDR < 0.25.

### Cell lines and human tissue samples

BC 4T1 cells were purchased from the American Type Culture Collection (ATCC) and cells were cultured in RPMI medium 1640 (GIBCO, Gaithersburg, MD, USA) supplemented with 10% fetal bovine serum (HyClone, Logan, USA) and 1% penicillin/streptomycin (Invitrogen, Waltham, MA). Cells were grown in 24-well culture dishes (VWR International; Radnor, PA) containing 1.0 ml cell culture medium at 37 °C in a Hera Cell 5% CO_2_ incubator (Thermo Fisher Scientific; Waltham, MA). Culture medium was replaced after 1 day of seeding and then every 48 h thereafter. A total of 60 formalin-fixed paraffin-embedded BC samples were collected from BC patients who underwent curative-intent surgery without prior radiotherapy and chemotherapy at the Department of Pathology of the Third Affiliated Hospital of Southern Medical University. Informed consent was obtained from each patient on the day of admission. The study was approved by the ethics committee of The Third Affiliated Hospital of Southern Medical University.

### Immunohistochemical analyses

Primary lesion and bone metastasis samples were fixed in formalin, embedded in paraffin, and analyzed by immunohistochemical analysis. Sections (2.5 μm) were deparaffinized and rehydrated, and endogenous peroxidase activity was inhibited with 0.3% H_2_O_2_-methanol solution. Samples were blocked in 5% normal goat serum for 1 h, probed with anti-CENPF antibodies (Affinity, DF2310, 1:50 dilution) and anti-PTHrP (ABclonal, A12492, 1:150 dilution) at 4 °C overnight, and labeled with biotinylated secondary antibodies. The immunoreaction signal was developed with DAB staining, and slides were counterstained in hematoxylin. Stained tissue sections were viewed under a light microscope (Nikon ECLIPSE Ni-U, Tokyo, Japan).

### Animal models

Six-week-old female BALB/c mice were purchased from the Central Laboratory of Animal Science of Southern Medical University (Guangzhou, China). Mice were provided with standard laboratory diet and drinking water ad libitum, and maintained in a pathogen-free environment at a constant temperature of 23 ± 1 °C and humidity of 55 ± 5% and with a 12-h light/12-h dark cycle. All studies for animals were reviewed and approved by the Institutional Animal Care and Use Committee of Southern Medical University. Mice were randomly divided into 2 groups: (1) To investigate the expression of CENPF in primary BC lesions, 1 × 10^5^ 4T1 cells were inoculated into the mammary fat pad of mice; (2) To investigate CENPF expression in bone metastasis of BC cells, 1 × 10^4^ 4T1 cells were inoculated into the left tibia of the mice. After 4 weeks, mice were sacrificed and their organs isolated. Collected organs were fixed in 10% neutralized formalin solution and paraffin embedded.

### siRNA transfection

CENPF siRNA (5′-GGAGATGCTTCAAACTCAA-3′) was obtained from RiboBio (Guangzhou, China) and transfected into 4T1 cells using lipofectamine 3000 (Thermo Fisher Scientific, Rockford, IL, USA). After 48 h, cells were harvested and assessed by western blot analysis.

### Western blotting

Cells were lysed in RIPA buffer (KeyGEN BioTECH) and quantified using Bradford Assays (KeyGEN BioTECH). Lysates were resolved on SDS–PAGE, and transferred to PVDF membranes (Millipore). Membranes were probed with primary antibodies overnight at 4 °C. The primary antibodies included anti-CENPF (Affinity, DF2310, 1:1000 dilution), anti-mTOR (proteintech, 20657-1-AP, 1:1000 dilution), anti-p-mTOR (Absci, AB11221, 1:1000 dilution), anti-AKT (Cell Signaling, #4691, 1:1000 dilution), anti-p-AKT (Cell Signaling, #13038, 1:1000 dilution), anti-PTHrP (ABclonal, A12492, 1:1000 dilution). Membranes were labeled with the appropriate HRP-conjugated secondary antibodies (Fdbio science, FDM007 or FDR007, 1:10,000 dilution) and chemiluminescence was detected using FDbio-Femto ECL western blotting detection reagents (Fdbio science, Hangzhou, China).

### Statistical analysis

Data are presented as the mean ± standard deviation (SD) from three independent assays using SPSS 22.0 (IBM SPSS Inc. Chicago, IL). A two-tailed Student’s t-test (two-tailed) was used to assess differences between the conditions. A *p*-value < 0.05 was considered statistically significant.

## Results

### CENPF is overexpressed in breast and lung cancer

**Fig. 1:**
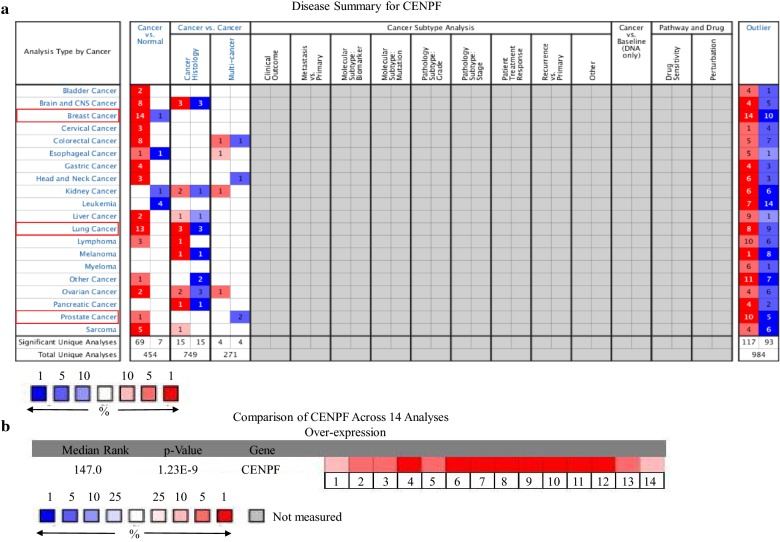
mRNA expression of CENPF in different tumors. Graphs show the number of datasets with statistically significant mRNA overexpression (red) or down-regulation (blue) of the target gene (cancer vs. normal tissue and cancer vs. cancer). *p* value thresholds were 0.01. Numbers in each cell indicate the number of analyses that met the threshold within each analysis and cancer type. Cell colors demonstrate the best gene rank percentile for analyses. CENPF was compared across 14 analyses. Values of the genes indicate the median rank. *p* values were assessed for each gene and for each median-ranked analysis (**a**, **b**)

**Fig. 2:**
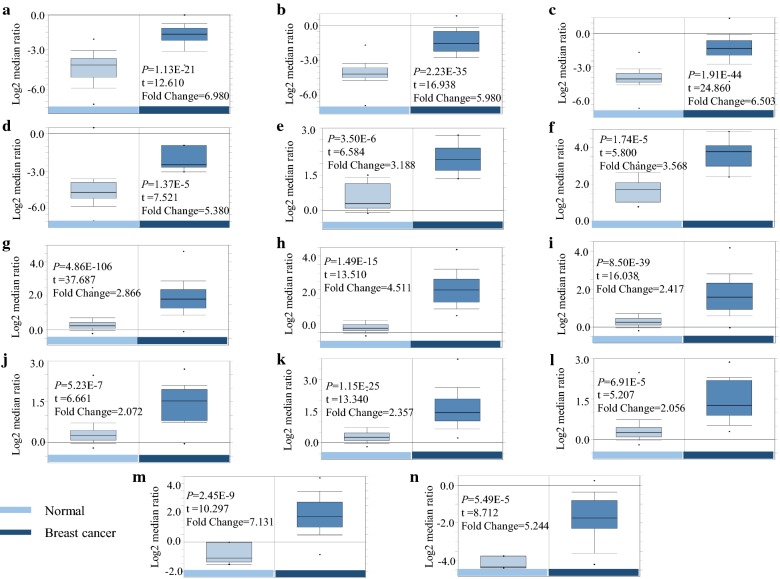
CENPF analysis in BC (*ONCOMINE* database). Box plots derived from gene expression data in *ONCOMINE* comparing the expression of CENPF in normal and BC tissue. *p* values were set at 0.01 and the fold change was set as 2. Comparison of CENPF mRNA expression in normal and BC tissue (**a**–**n**)

**Fig. 3:**
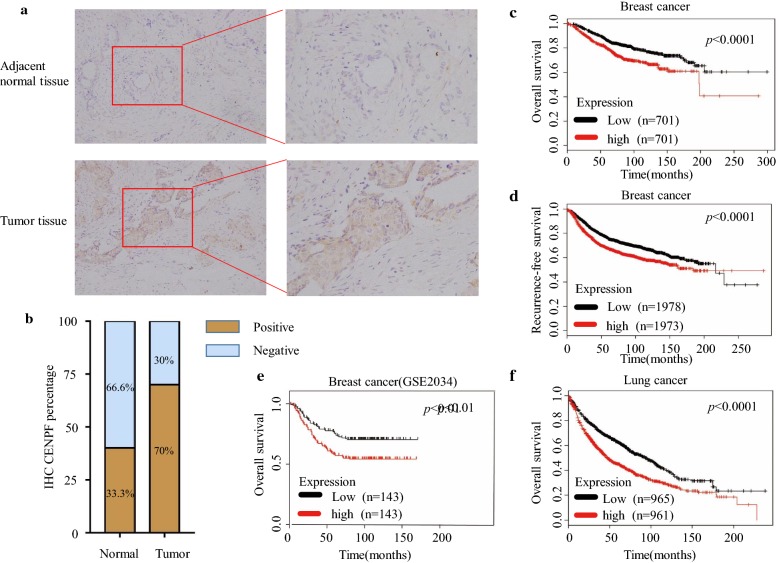
CENPF in tumor tissue and adjacent histologically normal tissue of BC patients (×400) (**a**). Percentage of CENPF IHC in BC and matched adjacent normal tissue. **b** High CENPF mRNA levels were associated with shorter OS (**c**), reduced RFS (**d**), and shorter OS in BC patients with high CENPF mRNA expression (**e**). High mRNA levels of CENPF were associated with shorter OS in lung cancer patients (**f**)

From ONCOMINE analysis, CENPF mRNA expression was significantly higher in BC samples across the 14 datasets in different cancer types (Table 1 and Fig. 1a, b). CENPF transcripts were elevated by ≥ 5.3-fold in BC samples compared to normal tissue. The samples included 593 samples derived from the TCGA (the Cancer Genome Atlas) database (Fig. 2a–d). In previous studies [20], CENPF was ≥ 3.1 fold elevated in BC samples compared to normal tissue (Fig. 2e, f). Similarly, CENPF was ≥ 2.0 fold elevated in 2136 BC samples [21] (Fig. 2g–l). In other studies [22, 23], CENPF was 5.2 fold (total samples = 47) and 7.1 fold higher in BC samples (Fig. 2m, n) compared to normal tissue. To further determine the role of CENPF in BC, 60 BC samples and paired normal tissue were collected and assessed by immunohistochemistry (IHC) staining. This confirmed that CENPF is expressed to higher levels in BC (42/60, 70%) compared to normal tissues (20/60, 33.3%) (*p *< 0.01) (Fig. 3a, b).

**Table 1:** The situation of each sub-database

Database	*P* value	Fold change	Sample size	Sample size of normal	Sample size of breast cancer
TCGA breast	1.13E−21	6.980	97	61	36
	2.23E−35	5.980	137	61	76
	1.91E−44	6.503	450	61	389
	1.37E−5	5.380	68	61	7
Ma breast 4	3.50E−6	3.188	23	14	9
	1.74E−5	3.568	23	14	9
Curtis breast	4.86E−106	2.866	1700	144	1556
	1.49E−15	4.511	176	144	32
	8.50E−39	2.417	292	144	148
	5.23E−7	2.072	165	144	21
	1.15E−25	2.357	234	144	90
	6.91E−5	2.056	158	144	14
Richardson breast 2	2.45E−9	7.131	47	7	40
Zhao breast	5.49E−5	5.244	41	3	38

Lung cancer is also prone to bone metastasis. Our analysis also demonstrated significantly higher CENPF expression in lung cancer versus normal samples (Additional file 1: Fig. S1A–I). In the datasets reported by Bhattacharjee and coworkers [24] from 186 samples, CENPF was 24.5 fold higher in lung cancer samples compared to normal tissue (Additional file 1: Fig. S1A).

### High CENPF mRNA expression correlates with poor OS and RFS in BC patients

Kaplan–Meier analysis demonstrated that high CENPF mRNA expression is significantly associated with shorter OS and RFS in BC (HR = 1.61 (1.3–2), *p *= 1.3e−05) and (HR = 1.39 (1.25–1.55), *p *= 3e−09 respectively), (Fig. 3c, d). The analysis of GSE2034 (from GEO datasets) demonstrated that high CENPF mRNA expression led to a poor prognosis (*p *= 0.0038) (Fig. 3e). Similarly, high CENPF mRNA expression was associated with decreased survival in lung cancer (HR = 1.57 (1.38–1.78), *p *= 3.4e−12) (Fig. 3f). Of note, high CENPF expression significantly correlated to shorter OS and RFS in BC patients. This indicated a role for CENPF in the prognosis of BC.

### CENPF expression is higher in bone metastasis in BC than that in primary BC lesions and other organs

**Fig. 4:**
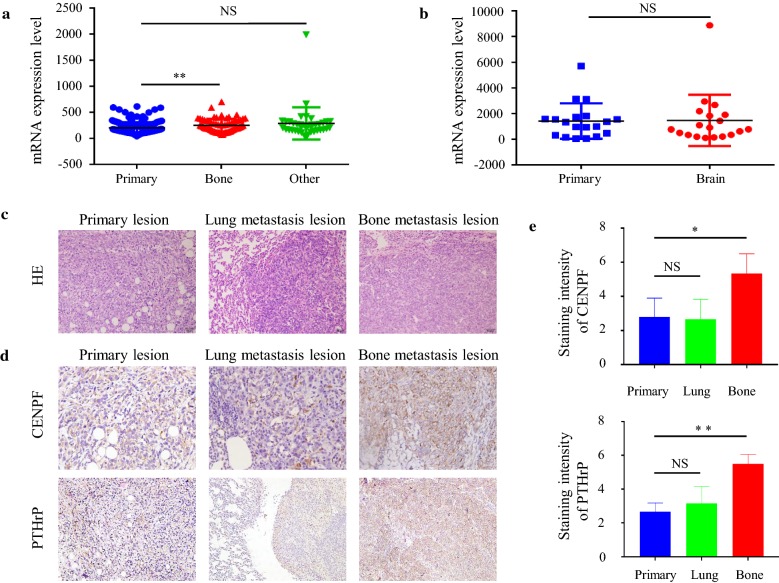
CENPF mRNA expression is higher in bone metastasis than primary BC lesions and other target organs (**a**). CENPF mRNA expression in primary lesions and brain metastasis does not differ (**b**). Histopathological diagnosis (H & E staining) of samples from primary lesions (×200), lung metastasis (×200), and bone metastasis (×200) of BALB/C mice 4T1 BC models (**c**). CENPF and PTHrP in primary lesions (×400), lung metastasis (×400) and bone metastasis (×400) of BALB/C mice 4T1 BC models (**d**). IHC staining intensity of CENPF and PTHrP is shown (**e**). Error bars represents the mean ± SD of three replicate samples. *p < 0.05, **p < 0.01

The GSE2034 is a published dataset consisting of 180 BC specimens without bone metastasis, 69 BC specimens with bone metastasis, and 37 BC specimens with other organ metastasis (including lung and brain metastasis). We compared the mRNA expression of CENPF in the 3 types of specimen and found that the expression of CENPF in bone metastasis is higher than primary BC lesions, but does not differ between primary BC lesions and the metastasis of other organs (Fig. 4a). Through GDS5306 analysis, published datasets consisting of 19 brain metastasis specimens and matched primary breast tumor specimens of 19 BC patients, we also found that the mRNA expression of CENPF does not differ between primary breast tumors and brain metastasis tissue (Fig. 4b).

In the four 4T1 primary BC models and four bone metastasis models of BALB/C mice, CENPF was expressed to higher levels in 75% (3/4) of bone metastasis tissue samples compared to primary lesions and lung metastasis tissues. Interestingly, the expression of PTHrP in these three different tissues showed the same trend as CENPF. However, no significant differences were observed between primary lesions and lung metastatic tissue (Fig. 4d). These results further demonstrate that CENPF plays an important role in bone metastasis during BC. In addition, there may be a close correlation between CENPF and PTHrP in breast cancer bone metastasis.

### GSEA reveals a potential role of CENPF in oncogenic Signaling during tumor metastasis

**Fig. 5:**
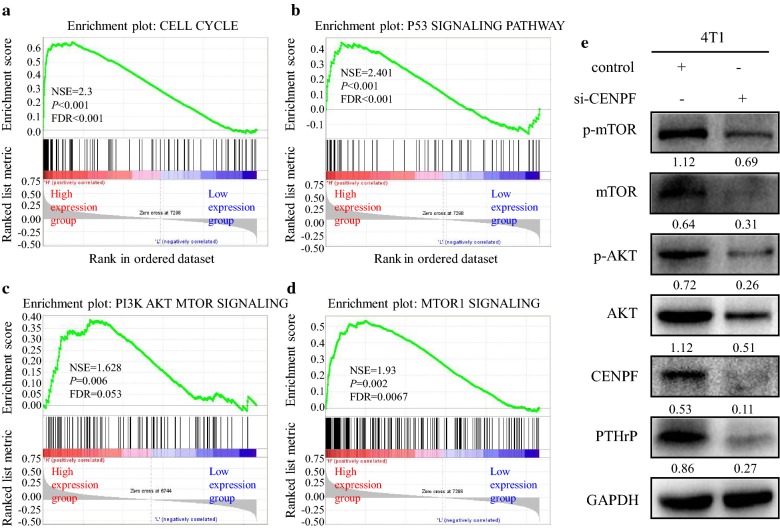
GSEA reveals potential downstream signaling of CENPF. GSEA results showed that the cell cycle (**a**), P53 signaling pathways (**b**), PI3K/AKT/mTOR signaling (**c**), and mTORC1 pathways (**d**) were enriched in the high CENPF expression group. Top panels indicate the enrichment scores for each gene. Bottom panels show the ranking metrics of each gene. Y-axis: ranking metric values; X-axis: ranks for all genes. NES: normalized enrichment score. CENPF silencing reduced the expression of AKT, p-AKT, mTOR, p-MTOR and PTHrP as assessed by western blot analysis (**e**)

To identify the cellular mechanisms by which CENPF influences tumor development, gene set enrichment analysis (GSEA) was used to compare the gene expression profiles of CENPF^low^ and CENPF^high^ in BC specimens. The GSE2034 database contains 286 BC tissues divided into CENPF^low^ (n = 143) and CENPF^high^ (n = 143) groups based on the median expression level of CENPF. GSEA analysis revealed a significant association between CENPF and cell cycle regulation, P53, and PI3K–AKT–mTOR signaling suggesting a role for these pathways in the metastatic activity of CENPF (Fig. 5a–d). As expected, the activation of AKT/mTOR signaling pathway and the expression of PTHrP were dramatically inhibited in 4T1 cells with silenced CENPF (Fig. 5e).

## Discussion

Previous studies have demonstrated that the overexpression of CENPF plays an important role in prostate cancer development [25, 26]. CENPF has been shown to be a synergistic master regulator of prostate cancer malignancy and a prognostic indicator of poor survival and metastasis [18]. However, the roles of CENPF in the other cancers are less well understood and the functions of CENPF remain undefined. In this study, we show that CENPF is upregulated in BC tissue, particularly in BC bone metastatic lesions, which positively correlates with poor survival in human BC patients. Furthermore, we performed GSEA to explore the potential mechanisms of CENPF bone metastasis.

Using bioinformatics and experimental analysis, CENPF was found to play a vital role in BC progression and bone metastasis. CENPF was found to be frequently upregulated in BC and other cancers prone to bone metastasis including lung cancer and prostate cancer (Figs. 1, 2). To determine the expression of CENPF in human BC tissue, we performed IHC staining of CENPF in 60 BC tissues. The staining intensity of CENPF was predominantly in BC tissues, and weak staining was detected in normal adjacent tissue (Fig. 3a, b). Secondly, we performed Kaplan–Meier analyses to show that CENPF is a prognostic marker for clinical outcomes (Fig. 3c–f). Thus, CENPF may function as a tumor promoter during BC progression.

Additionally, we used GEO datasets containing primary BC lesions and distant metastatic lesions to perform microarray analysis. We found that CENPF expression is higher in bone metastatic lesions compared to primary BC lesions and other distant organs (Fig. 4a, b). As CENPF is highly expressed in BC, particularly in bone metastatic lesions, these results are consistent with its reported role in bone metastasis in prostate and lung cancer cases.

Given these findings, we hypothesized that CENPF is closely related to bone metastasis in BC. Furthermore, we performed IHC staining of CENPF in BC primary lesions, lung metastasis lesions, and bone metastasis lesions, which were collected from our animal model. We found that the intensity of CENPF staining was higher in primary lesions and lung metastasis lesions (Fig. 4d, e). These results confirm that CENPF promotes BC bone metastasis.

Since bone metastasis is the leading cause of BC related death [27, 28], understanding the molecular role of CENPF driven bone metastasis can direct future therapeutic strategies. The development of BC bone metastasis is a complex process involving crosstalk between disseminated BC cells and bone-derived molecules, leading to deregulated signaling pathways that are critical for normal bone remodeling processes [4]. Herein, we performed GSEA to explore the potential mechanisms of CENPF driven BC progression and bone metastasis. Our results showed that the CENPF expression was significantly associated with P53, cell-cycle progression and the G2 M-Checkpoint. Previous studies have shown that CENPF is a component of the nuclear matrix during the G2 stage of interphase, where in gradually accumulates during the cell cycle, reaching peak levels in the G2/M phase, and is degraded upon the completion of mitosis [29]. Notably, CENPF expression was also enriched in the PI3K–AKT–mTOR and mTORC1 signaling pathways and we confirmed that the activation of AKT/mTOR signaling pathway and the expression of PTHrP were dramatically inhibited in 4T1 cells with silenced CENPF (Fig. 5). As we have discovered, knocking down CENPF not only inhibits the synthesis of mTOR and AKT, but also inhibits their phosphorylation (Fig. 5e).

PI3K/AKT/mTOR signaling is an important intracellular pathway that is frequently activated in diverse cancers. PI3K/AKT/mTOR regulates cell proliferation, differentiation, cellular metabolism and cancer cell survival. PI3K/AKT/mTOR activation promotes tumor development as drug resistance [30, 31]. Cancer bone metastasis is a complex, multistage process that includes local invasion, intravasation, survival in the circulation, extravasation, and colonization [32, 33]. Within this process are various molecules including parathyroid hormone-related peptide (PTHrP) [11, 12]. Previous studies have demonstrated that the downstream S6 kinase 1 of mTORC1 interacts with and phosphorylates Gli2, permitting its release and the subsequent transcriptional activation of PTHrP, a key regulator of bone development [34]. PTHrP participates in bone remodeling through osteoclastogenesis and facilitates tumor localization and growth in the bone [35]. CENPF overexpression in BC may thus activate mTORC1 to regulate PTHrP, which modifies the host osseous environment to promote osteoclast formation and bone colonization [36].

**Fig. 6:**
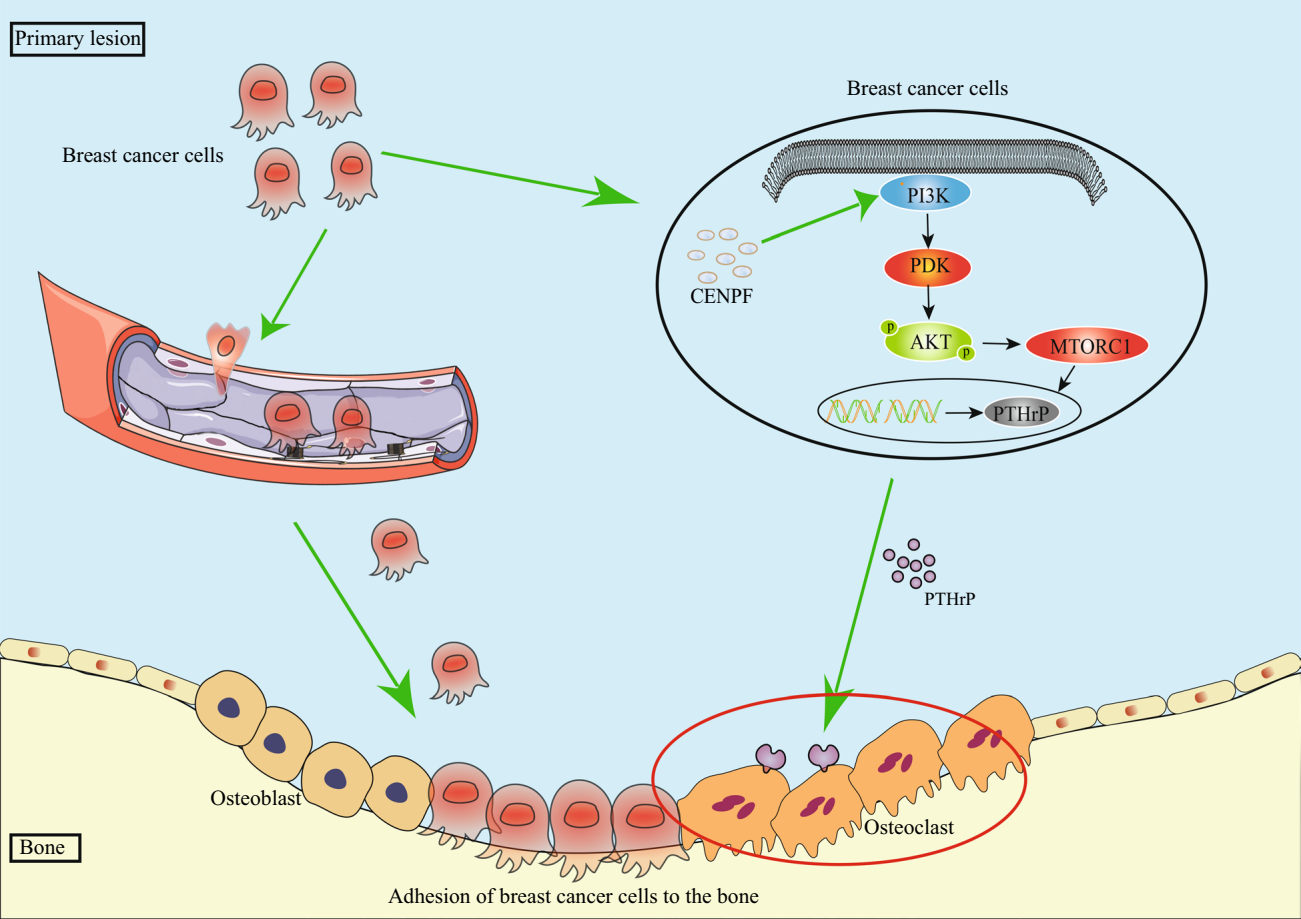
CENPF regulates PI3K/AKT/MTORC1 signaling in BC bone metastasis. In BC cells, CENPF activates PI3K/AKT/mTORC1 signaling resulting in increased PTHrP secretion. This modifies the host osseous environment to promote osteoclast formation and bone colonization

In summary, we have revealed the metastatic promoter function of CENPF in BC progression and bone metastasis. High CENPF expression in BC activates mTORC1 and regulates PTHrP, which modifies the bone microenvironment permitting an ease of transfer of BC cells to the bone (Fig. 6). However, some limitations remain as the potential molecular mechanisms were not verified experimentally. We envision that therapeutic intervention centered on inhibiting CENPF function could be useful for the prevention of BC bone metastasis.

## Conclusion

Our results demonstrated that CENPF promotes BC bone metastasis by activating the PI3K–AKT–mTORC1 signaling pathway. CENPF may serve as a novel therapeutic, diagnostic, and/or prognostic target in breast cancer treatment.

## Supplementary information

**Additional file 1: Figure S1.** CENPF in lung cancer (*ONCOMINE* database). Box plots derived from gene expression data in *ONCOMINE* comparing the expression of the CENPF in normal and LC tissue. *p-*values were set at 0.01 and the fold change was defined as 2. Comparison of CENPF mRNA expression in normal and lung cancer tissue (A–L).

## Data Availability

Datasets used and/or analyzed data are available from the corresponding author upon reasonable request.

## Abbreviations

CENPF: centromere protein F
BC: breast cancer
IHC: immunohistochemical
HE: hematoxylin
GEO: the Gene Expression Omnibus
OS: overall survival
RFS: relapse free survival
GSEA: gene set enrichment analysis
PTHrP: parathyroid hormone-related peptide
p: phosphor
PI3K: phosphatidylinositol 3-kinase
AKT: serine–threonine protein kinase
mTOR: mechanistic target of rapamycin kinase
mTORC1: mechanistic target of rapamycin kinase complex 1
BM: bone metastasis
IL-8: interleukin 8
FOXM1: forkhead box protein M1
VCAM-1: vascular cell adhesion molecule 1
HCC: hepatocellular carcinoma
COUP-TFII: chicken ovalbumin upstream promoter transcription factor 2
PC: prostatic cancer
NES: normalized enrichment score
FDR: false discovery rate
MSigDB: Molecular Signatures Database
ATCC: the American Type Culture Collection
SD: standard deviation
HR: hazard ratio
