# Supplementary figures and images for: Overexpression of CENPF correlates with poor prognosis and tumor bone metastasis in breast cancer
Source: Cancer Cell Int. 2019 Oct 11;19:264. doi: 10.1186/s12935-019-0986-8 (PMC6788011; doi:10.1186/s12935-019-0986-8)

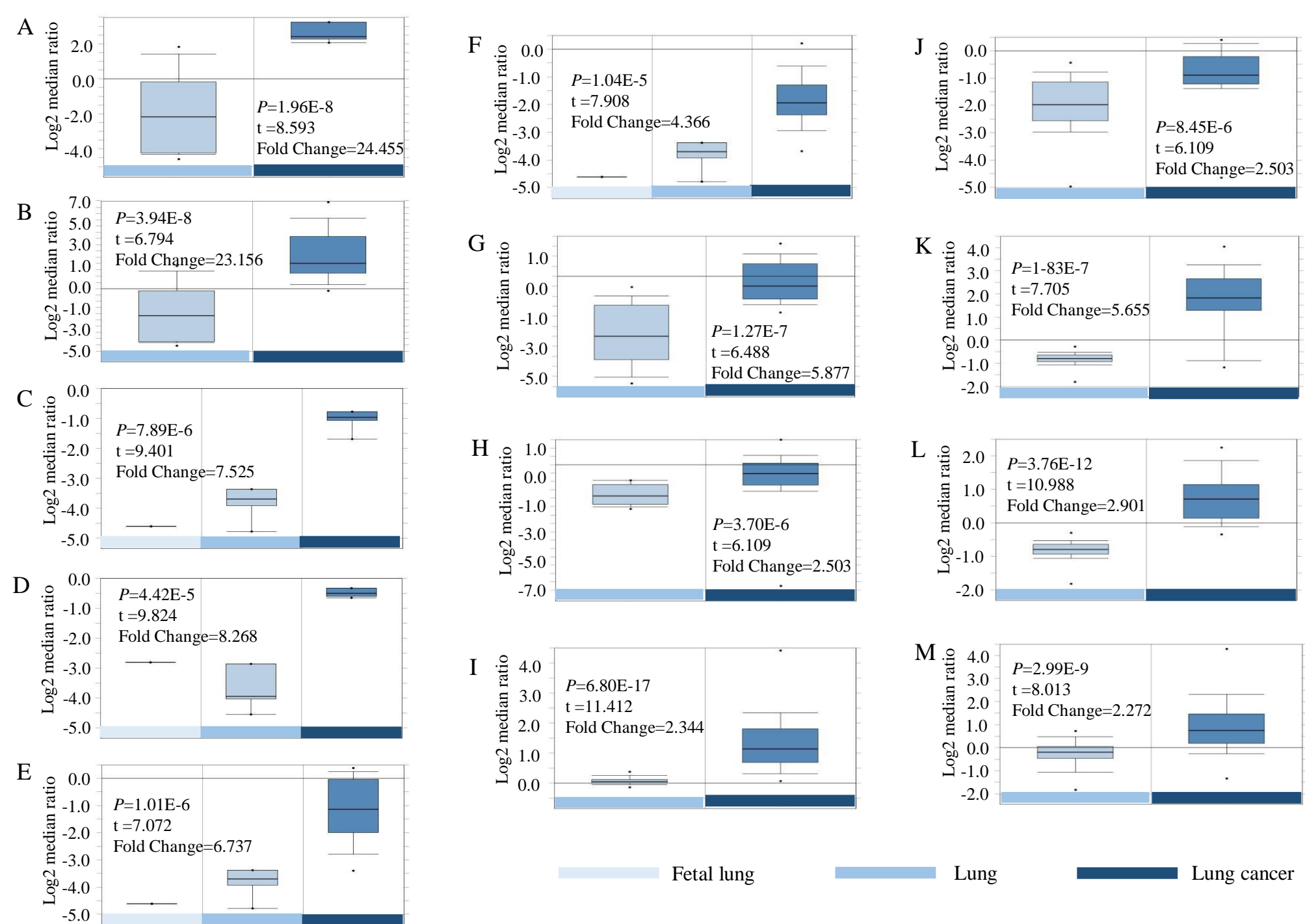

Supplement: Supplementary file 1 — Additional file 1: Figure S1. CENPF in lung cancer (ONCOMINE database). Box plots derived from gene expression data in ONCOMINE comparing the expression of the CENPF in normal and LC tissue. p-values were set at 0.01 and the fold change was defined as 2. Comparison of CENPF mRNA expression in normal and lung cancer tissue (A–L). [file 12935_2019_986_MOESM1_ESM.pdf]
